# Ischemic Infarction of Pituitary Apoplexy: A Retrospective Study of 46 Cases From a Single Tertiary Center

**DOI:** 10.3389/fnins.2021.808111

**Published:** 2022-01-24

**Authors:** Qiang Zhu, Yuchao Liang, Ziwen Fan, Yukun Liu, Chunyao Zhou, Hong Zhang, Tianshi Li, Yanpeng Zhou, Jianing Yang, Yinyan Wang, Lei Wang

**Affiliations:** Department of Neurosurgery, Beijing Tiantan Hospital, Capital Medical University, Beijing, China

**Keywords:** pituitary apoplexy, ischemic infarction, coagulative necrosis, pituitary ring sign, ghost cells

## Abstract

**Objective:**

Ischemic infarction of pituitary apoplexy (PA) is a rare type of pituitary apoplexy. This study aims to characterize ischemic PA via clinical presentations, imaging data, histopathological manifestations, and focus on the management and prognosis of the disease.

**Methods:**

This study retrospectively identified 46 patients with ischemic PA confirmed using histopathology at a single institution from January 2013 to December 2020. The clinical presentations, imaging data, laboratory examination, management, and outcomes were collected. We then summarized the clinical presentations, imaging features, intraoperative findings, and histopathological manifestations, and compared the outcomes based on the timing of surgical intervention.

**Results:**

Headache was the most common initial symptom (95.65%, 44/46), followed by visual disturbance (89.13%, 41/46), and nausea and vomiting (58.70%, 27/46). 91.3% of the patients had at least one pituitary dysfunction, with hypogonadism being the most common endocrine dysfunction (84.78%, 39/46). Cortisol dysfunction occurred in 24 (52.17%) patients and thyroid dysfunction occurred in 17 (36.96%). Typical rim enhancement and thickening of the sphenoid sinus on MRI were seen in 35 (85.37%) and 26 (56.52%) patients, respectively. Except for one patient with asymptomatic apoplexy, the remaining patients underwent early (≤ 1 week, 12 patients) and delayed (> 1 week, 33 patients) transsphenoidal surgery. Total tumor resection was achieved in 27 patients and subtotal tumor resection in 19 patients. At surgery, cottage cheese–like necrosis was observed in 50% (23/46) of the patients. At the last follow-up of 5.5 ± 2.7 years, 92.68% (38/41) of the patients had gained a significant improvement in visual disturbance regardless of surgical timing, and 65% of the patients were still receiving long-term hormone replacement therapy.

**Conclusion:**

Patients with ischemic PA can be accurately diagnosed by typical imaging characteristics preoperatively. The timing of surgical intervention does not significantly affect the resolution of neurological and endocrinological dysfunctions. Preoperative endocrine dysfunctions are common and usually appear to be poor after surgical intervention.

## Introduction

Pituitary apoplexy (PA) is a rare, life-threatening emergency caused by hemorrhage and/or ischemia of a preexisting pituitary adenoma, and is most often a clinically non-functioning macroadenoma (Briet et al., 2015). According to epidemiological studies, the prevalence of pituitary apoplexy is about 6.2 cases per 100,000 people. Approximately 2–12% of patients with various types of pituitary adenoma experience apoplexy (Wakai et al., 1981; McFadzean et al., 1991; Bonicki et al., 1993; da Motta et al., 1999; Randeva et al., 1999; Ayuk et al., 2004; Verrees et al., 2004; Liu and Couldwell, 2006; Dubuisson et al., 2007; Murad-Kejbou and Eggenberger, 2009; Turgut et al., 2010; Möller-Goede et al., 2011), and more than 75% of apoplexy occurs in patients with an undiagnosed pituitary adenoma (Randeva et al., 1999; Biousse et al., 2001; Chacko et al., 2002; Ayuk et al., 2004; Sibal et al., 2004; Gruber et al., 2006; Dubuisson et al., 2007; Liu et al., 2010; Leyer et al., 2011; Möller-Goede et al., 2011; Pal et al., 2011; Sarwar et al., 2013; Bujawansa et al., 2014; Kinoshita et al., 2014). The clinical presentations of pituitary apoplexy vary, including sudden headache, nausea and vomiting, ophthalmic dysfunction, fever, altered mental status, and even death. The pathophysiological mechanism of PA remains unclear. There have been numerous reports on the diverse predisposing factors of pituitary apoplexy, such as head trauma, pregnancy, diabetes mellitus, hypertension, anticoagulant medications, dynamic study of the pituitary gland, and surgeries (Möller-Goede et al., 2011). Compared with symptomatic pituitary apoplexy, approximately 25% of patients experienced asymptomatic pituitary apoplexy (Wakai et al., 1981; Fraioli et al., 1990; Onesti et al., 1990; Bonicki et al., 1993; Kinoshita et al., 2014).

Hemorrhage has been known as the primary cause of pituitary apoplexy. A pure ischemic infarction of pituitary apoplexy is rarely reported compared with hemorrhage. With the limited numbers of ischemic PAs reported in the literature, there is a poor understanding of the pathophysiology of this entity. This study reported the largest sample size of the patients presenting with a pure ischemic PA. Herein, we summarized the clinical presentations, endocrine function, and imaging data of patients with ischemic PA. Moreover, we further explored the effect of surgical timing on neuro-ophthalmic and endocrine outcomes. We hope that this study can provide a more comprehensive understanding and optimize the management of ischemic PA.

## Materials and Methods

We retrospectively analyzed patients with ischemic PA confirmed by pathology findings within the Beijing Tiantan Hospital from January 2012 to December 2020. The inclusion criteria are as follows: (1) The patient was diagnosed with a pituitary adenoma by cranial CT or/and MRI; (2) postoperative pathological examinations confirmed patients with pituitary apoplexy and only exhibited an ischemic infarction; (3) patients had complete clinical data. The medical records of index hospitalization and the last clinical visit were reviewed to obtain information on demographic data, clinical presentation, laboratory examination, neuro-ophthalmic evaluation, imaging data, management profile, and pathological manifestations. The baseline characteristics of the included patients are described in Table 1.

**TABLE 1:** Baseline characteristics of among 46 patients with ischemic PA.

Clinical factors	Values
Age (yr)	46.78 ± 12.32
Sex (M/F)	35M/11F
**Symptoms and sign**	
Headache	95.65% (44/46)
Nausea and vomiting	58.70% (27/46)
Hyponatremia	39.13% (18/46)
Diabetes insipidus	10.87% (5/46)
Fever	10.87% (5/46)
**Neuro-ophthalmic examination**	
Decrease visual acuity	89.13% (41/46)
Visual field deficit	82.61% (38/46)
Ocular palsy	50.00% (23/46)
**Endocrine dysfunction**	
Hypocorticolism	52.17% (24/46)
Hypothyroidism	36.96% (17/46)
Hypogonadism	84.78% (39/46)
Corticotropic deficiency or secondary hypothyroidism	58.70% (27/46)
Corticotropic deficiency and secondary hypothyroidism	30.43% (14/46)
**Imaging characteristics**	
Maximal tumor diameter (mm)	26.54 ± 6.03
Knosp classification (1–2 vs. 3–4)	28 vs. 14
Pituitary ring sign	85.37% (35/41)
Thickening of the sphenoid sinus mucosa	56.52% (26/46)
Ischemic imaging	23.91% (11/46)
**Treatment**	
Surgery (early vs. delayed)	12 vs. 33
EOR (Total resection vs. Subtotal resection)	27 vs. 19
**Histology**	
NFPA vs. FPA	36 vs. 6
**Follow-up**	
Duration (m)	66.29 ± 32.85
Recurrence	2

*NFPA, non-functioning pituitary apoplexy; FPA, functioning pituitary apoplexy; yr, year; M, male; F, female; mm, millimeter; m, month; EOR, extent of resection.*

Endocrinological assessments primarily included thyroid-stimulating hormone (TSH) and thyroid hormone levels, growth hormone (GH), insulin-like growth factor (IGF), luteinizing hormone (LH), follicle-stimulating hormone (FSH), testosterone, and random cortisol. Diabetes insipidus (DI) was diagnosed when serum osmolality was > 295 mOsm/kg, whereas a corresponding urine osmolality was < 300 mOsm/kg in fluid deprivation tests in the case of polyuria and polydipsia, and a subsequent response to arginine vasopressin was observed. Prolactin deficiency or excess was determined according to a clinical reference range. At admission, random serum cortisol levels of < 50 nmol/L (range 50–250 mmol/L) indicated hypocortisolism. Low free T4 together with low or inappropriate normal TSH was considered secondary hypothyroidism. Gonadotropic deficiency in men and premenopausal women was defined as low testosterone levels with having low or inappropriately normal gonadotrophin levels, and in postmenopausal women, it was defined as inappropriately low gonadotrophin levels for menopausal age. Ophthalmologic evaluations included assessments of binocular visual acuity, visual field, and cranial nerve functions and were performed by an experienced ophthalmologist.

Two experienced radiologists evaluated all imaging data. The tumor size was classified as microadenoma (<1 cm), macroadenoma (1–4 cm), and giant pituitary adenoma (>4 cm). The relationship between the tumor and the cavernous sinus was evaluated according to knosp criteria, and patients with knosp grades 3 and 4 (tumor invasion beyond a line tangential to the lateral margins of the cavernous internal carotid artery, and total internal carotid artery encasement, respectively) were defined as positive cavernous sinus invasion. Based on the interval from initial onset to severe symptoms such as visual disturbance, the patients with ischemic PA were classified as acute onset (<3 days), subacute onset (3–14 days), and chronic onset (>14 days) (Xiao et al., 2015). All patients finally underwent transsphenoidal surgery (TSS) by experienced neurosurgeons. Early surgery was defined as an operation performed within 7 days of symptom onset, whereas delayed surgery was defined as an operation beyond 7 days of symptom onset. All patients were followed up for more than 6 months. On the last follow-up, the requirement of hormone replacement was considered pituitary dysfunction. The comparison of clinical presentations and outcomes of ischemic PA between early and delayed surgery groups is summarized in Table 2.

**TABLE 2:** Comparison of clinical presentation and outcomes of ischemic PA between early and delayed surgery groups.

	Early surgery (*n* = 12)	Delayed surgery (*n* = 33)	*P*-value
Age* (yr)	46.58 ± 14.34	46.30 ± 11.65	0.864
Sex**^∧^** (M/F)	11M/1F	24M/9F	0.344
**Symptoms and sign**			
Headache**^∧^**	100% (12/12)	96.97% (32/33)	1
Nausea and vomiting**^∧^**	66.67% (8/12)	57.58% (19/33)	0.836
Hyponatremia**^#^**	50.00% (6/12)	63.64% (21/33)	0.409
**Ophthalmic examination**			
Decrease visual acuity**^&^**	91.67%% (11/12)	90.91% (30/33)	1
Visual field deficit**^∧^**	83.33% (10/12)	51.52% (17/33)	0.114
Ocular palsy**^#^**	41.67% (5/12)	54.55% (18/33)	0.445
**Endocrine dysfunction**			
Hypocorticolism**^#^**	50.00% (6/12)	54.55% (18/33)	0.787
Hypothyroidism**^∧^**	16.67% (2/12)	45.45% (15/33)	0.157
Hypogonadism**^∧^**	83.33% (10/12)	84.85% (28/33)	1
Corticotropic deficiency or secondary hypothyroidism**^#^**	50.00% (6/12)	63.64% (21/33)	0.409
Corticotropic deficiency and secondary hypothyroidism**^∧^**	16.67% (2/12)	36.36% (12/33)	0.369
**Imaging characteristics**			
Maximal tumor diameter* (mm)	27.08 ± 5.089	25.70 ± 5.193	0.430
Knosp classification**^#^** (1–2 vs. 3–4)	(5 vs. 6)	(7 vs. 24)	0.049
Pituitary ring sign**^&^**	100% (11/11)	79.31% (23/29)	0.162
Thickening of sphenoid sinus mucosa**^∧^**	83.33% (10/12)	53.33% (16/33)	0.080
**Clinical outcomes**			
Total improved vision**^∧^**	90.91% (10/11)	82.14% (23/28)	0.850
Total improved ocular palsy	100% (5/5)	100% (16/16)	–
Improved hypocortisolism	20% (1/5)	13.33% (2/15)	–
Improved hypothyroidism	0% (0/2)	35.71% (5/14)	–
EOR**^∧^** (Total resection vs. Subtotal resection)	1 vs. 11	19 vs. 14	0.009
Follow-up (m)	72.11 ± 28.52	64.51 ± 34.90	0.504

*P-value calculated by t-test.

*^#^P-value calculated by Pearson Chi-Square.*

*^∧^P-value calculated by Continuity Correction.*

*^&^P-value calculated by Fisher’s Exact Test.*

Continuous variables with normal distribution were described as means and SD, and categorical variables as medians and range. A *t*-test (normal distribution) or a Mann–Whitney rank-sum test was used when two sets of continuous variables were compared. Categorical variables were tested using a χ^2^-test. A two-sided *p* < 0.05 was considered statistically significant. The results were graphically represented when deemed necessary. SPSS version 22 (IBM Corp, Armonk, New York, United States) was used for statistical analysis.

## Results

### Patient Demographics

Table 1 summarizes the baseline characteristics of the patients in detail. A total of 46 patients were diagnosed with ischemic PA, including 35 males and 11 females, with a male-to-female ratio of more than 3:1. At diagnosis, the mean age was 46.78 years (*SD* = 12.32 years) ranging from 25 to 76 years. Compared with functioning pituitary adenoma, patients with non-functioning pituitary adenoma were significantly older (*p* = 0.038, *t*-test) (Table 3). The patients were mainly in their 40–50 s. Twenty patients had acute onset, 13 patients had subacute onset, and 12 had chronic onset. The median duration from symptom onset to the diagnosis of ischemic PA was 20 days (range, 3–90 days). Eleven patients had at least one or more possible predisposing factors, including diabetes mellitus, hypertension, and antiplatelet medications. Six patients suffered hypertension, six patients suffered diabetes mellitus, two patients suffered diabetes mellitus and hypertension, and one patient was using antiplatelet therapy due to a history of cerebral infarction. Dopamine receptor agonists like bromocriptine, cabergoline, and somatostatin analogs were not seen in our study.

**TABLE 3:** Comparison of clinical presentation and outcomes of ischemic PA between NFPA and FPA groups.

Clinical factors	NFPA (*n* = 36)	FPA (*n* = 36)	*P*-value
Age* (yr)	47.56 ± 12.25	36.33 ± 8.45	0.038
Sex (M/F)^&^	29M/7F	4M/2F	0.593
**Symptoms and sign**			
Headache^&^	97.22% (35/36)	83.33% (5/6)	0.268
Nausea and vomiting^&^	58.33% (21/36)	28.57% (2/6)	0.384
**Ophthalmic examination**			
Decrease visual acuity ^&^	97.22% (35/36)	66.67% (4/6)	0.049
Visual field deficit^&^	66.67% (24/36)	50.00% (3/6)	0.649
Ocular palsy^∧^	50.00% (18/36)	16.67% (1/6)	0.282
**Endocrine dysfunction**			
Hypocortisolism^&^	52.78% (19/36)	33.33 (2/6)	0.663
Hypothyroidism^∧^	41.67% (15/36)	16.67% (1/6)	0.476
Hypogonadism^&^	80.56% (29/36)	100% (6/6)	0.567
Corticotropic deficiency or secondary hypothyroidism^&^	58.33% (21/36)	33.33% (2/6)	0.384
Corticotropic deficiency and secondary hypothyroidism^∧^	33.33% (12/36)	16.67% (1/6)	0.733
**Imaging characteristics**			
Maximal tumor diameter*(mm)	26.25 ± 5.26	27.67 ± 10.33	0.603
Knosp classification^&^	81.25% (26/32)	100% (6/6)	0.562
Pituitary ring sign^&^	63.33% (19/30)	85.71% (6/7)	0.389
Thickening of sphenoid sinus mucosa^&^	57.58% (20/36)	71.43% (4/6)	0.685
Ischemic imaging^∧^	34.62% (9/36)	14.29% (1/6)	1
**Treatment**			
Surgery (early vs. delayed)^∧^	32.35% (11/36)	16.67% (1/5)	1
**Clinical outcomes**			
Total improved vision^&^	84.85% (28/33)	100% (4/4)	1
Total improved ocular palsy	100% (18/18)	100% (1/1)	–
Improved hypocortisolism	6.25% (1/16)	0% (0/1)	–
Improved hypothyroidism	23.08% (3/13)	0% (0/1)	
EOR^&^ (Total resection: Subtotal resection)	14:22	4:2	0.375
Follow-up* (m)	68.43 ± 31.57	79.32 ± 33.36	0.442

*P-value calculated by t-test.

*^#^P-value calculated by Pearson Chi-Square.*

*^∧^P-value calculated by Continuity Correction.*

*^&^P-value calculated by Fisher’s Exact Test.*

Headache was the most common symptom. All but one patient presented with a headache, including 21 patients who suffered sudden-onset headache. Among the patients with headaches, 27 patients were accompanied by nausea and vomiting. There were no patients with alerted mental status. Decreased vision was observed in 41 patients, and 4 patients were nearly blind. Thirty-eight patients presented with temporal hemianopia, and 23 patients experienced ophthalmoplegia. Fifteen patients had temporal hemianopia and oculomotor nerve paralysis. Five patients had a fever and 5 patients suffered from polydipsia and polyuria preoperatively.

### Laboratory Examination

Pituitary dysfunctions were common in this cohort. Forty-two (42/46, 91.30%) patients had one or more pituitary dysfunctions. Hypogonadism is the most common pituitary dysfunction in this study (84.78%, 39/46). At admission, low random cortisol levels and secondary hypothyroidism were seen in 24/46 (52.17%) and 17/46 (36.96%), respectively. Fourteen patients suffered hypocortisolism combined with secondary hypothyroidism. There was no significant difference in endocrine dysfunctions between the early and delayed surgery groups (Table 2), as well as in the functioning pituitary adenoma (FPA) and non-functioning pituitary adenoma (NFPA) groups (Table 3). Six patients had mild hyperprolactinemia, and the levels of prolactin did not exceed 2 times the upper limit. Nineteen patients had low serum prolactin. Hyponatremia occurred in 17 patients, including 10 cases (135–130 mmol/L), 5 cases (125–130 mmol/L), and 2 cases (< 125 mmol/L) of serum sodium.

### Neuroimaging Findings

**FIGURE 1:**
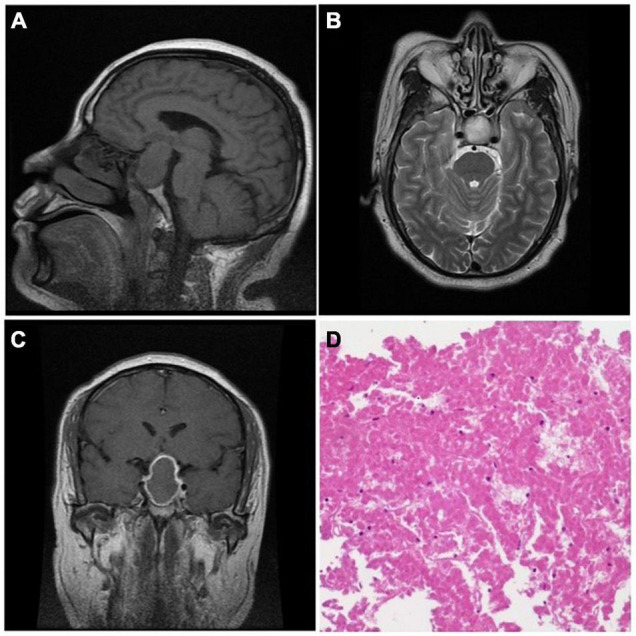
The MRI showed iso-intensity in T1-weighted imaging (**A**, sagittal) and hyperintensity in T2-weighted imaging (**B**, axial). After gadolinium administration, the imaging showed central iso-intensity with an enhanced rim (**C**, coronal). Histological manifestation **(D)** showed a large area of red massive coagulative necrosis without cellular structure (H&E ×100).

In this study, there were 45 macroadenomas and 1 giant adenoma. The mean diameter of the tumor was 26.54 mm (*SD* = 6.03 mm) ranging from 14 to 48 mm. In terms of tumor invasiveness, 29 and 14 patients were identified as knosp 1–2 and knosp 3–4, respectively (Table 1). There was a significant difference in tumor invasiveness between the early and delayed surgery groups (*p* = 0.049, Pearson χ^2^) (Table 2). In addition, 83.33% (35/42) of the patients showed iso-intensity in T1-weighted and hyperintensity in T2-weighted imaging (Figures 1A,B). After gadolinium administration, 87.5% (35/40) of the patients showed rim enhancement, also known as the pituitary ring sign (Figure 1C). Sixty-five percent (26/40) of the patients showed thickening of the sphenoid sinus on MRI. Eleven patients had cerebral ischemia lesions on MRI. Among these patients, two patients showed bilateral frontal subcortical ischemia on MRI, two patients had a lacunar infarction in the basal ganglia, three patients showed multiple lacunar infarct lesions, two patients had white matter ischemic lesions on MRI, and two patients showed ischemic demyelinating lesions.

### Intraoperative Findings and Pathology

At surgery, 96.52% (42/46) of the patients had solid tumor masses with poor or medium blood supply. The tumors were characterized by a grayish-yellow or a white-yellow mass with a soft texture or/and fibrous tissue, which is different from a classic pituitary adenoma. H&E staining of the tumor specimens showed massive acellular coagulative necrosis without any hemorrhagic changes under the microscope (Figure 1D). “Ghost cells” are a typical manifestation characterized by ghost outlines and no cellular structure. Moreover, white-yellow cottage cheese–like tissue was often seen during the operation (50%, 23/46). Four tumor samples lacked enough viable tumor tissue to make a biochemical diagnosis. Of the remaining 42 patients, there were 36 non-functional adenomas, 4 prolactinomas, 1 mixed prolactinoma/somatotrophinoma, and 1 TSH adenoma.

### Treatment and Prognosis

Four patients initially presented with mild headaches and transient double vision. They received watchful observation and supportive management. The symptoms were temporarily relieved; however, they progressed into a visual disturbance in later observations. These patients finally underwent TSS. The other 42 patients received TSS after diagnosis of their initial symptoms. The remaining 12 patients received early surgical intervention (≤ 7 days) and 33 patients received delayed surgical intervention (> 7 days), except for one patient with asymptomatic apoplexy. Total tumor resection was performed in 27 patients and subtotal tumor resection in 19 patients. Patients who received delayed surgical intervention significantly obtained a higher rate of total tumor resection compared with early surgery group (*p* = 0.009, continuity correction). There were no obvious postoperative complications. The mean follow-up duration was 66.29 months (*SD* = 32.85 months) ranging from 7.83 to 115 months (Table 1). Complete remission of decreased vision was seen in 10 out of 11 patients undergoing early surgery vs. 23 of 28 undergoing delayed surgery (*p* = 0.850, continuity correction) (Table 2). The duration of recovery ranged from 1 week to 6 months, with the majority of recovery at 3 months (78.95%, 30/38). The patients with ocular palsy recovered first among the ophthalmological dysfunctions, with 50% of the patients having significantly recovered before discharge. The rest of the patients had recovered completely during the 3-month follow-up (Table 4).

**TABLE 4:** Baseline characteristics of patients with ischemic PA between preoperative and postoperative.

	Preoperative	Postoperative
**Symptoms and sign**		
Headache	95.65% (44/46)	0
Hyponatremia	36.96% (17/46)	0
Diabetes insipidus	10.87% (5/46)	0
**Neuro-ophthalmic examination**		
Decrease visual acuity	89.13% (41/46)	15.91% (7/44)
Visual field deficit	60.87% (28/46)	11.11% (3/27)
Ocular palsy	50.00% (23/46)	0
**Endocrine dysfunction**		
Hypocortisolism	52.17% (24/46)	65.00% (26/40)
Hypothyroidism	36.96% (17/46)	46.34% (19/41)
Hypogonadism	82.61% (38/46)	63.16% (24/38)

After TSS, preoperative cortisol dysfunction was restored in three patients and secondary hypothyroidism in five patients. However, seven patients had postoperative new-onset cortisol dysfunction and five had postoperative secondary hypothyroidism during the last follow-up. Fifteen patients suffered postoperative new-onset polydipsia and polyuria and quickly recovered. The five patients with preoperative diabetes insipidus were completely cured after the operation, with the recovery duration ranging from 2 to 12 months. There was no significant difference in the prognosis of pituitary function between the early and delayed surgery groups (Table 2). Hormone replacement therapy was performed in the patients with long-term pituitary dysfunction, with testosterone only being administered to the men. As shown in Table 1, total tumor resection was achieved in 27 patients and subtotal tumor resection in 19 patients. Moreover, patients who received delayed surgery achieved a significantly higher rate of total tumor resection (*p* = 0.009, continuity correction) (Table 2). At the last follow-up, two patients revealed tumor recurrence through MRI examination and underwent TSS again. The prognosis was satisfactory without any severe complications.

## Discussion

Ischemic pituitary apoplexy, a rare type of pituitary apoplexy, shows unique intraoperative findings, imaging characteristics, and histological manifestations, different from hemorrhagic PA. There is a limited understanding of ischemic PA with a small number of reported cases. This study reported the largest number of ischemic PA patients confirmed by histopathology. The summarized clinical manifestations, imaging data, and intraoperative findings are further discussed as well as the effect of surgical timing on the prognosis of ischemic PA.

The average age of the patients in this study was 46.78 years, and the patients were mainly in the 40–50 s, which is similar to the pituitary apoplexy findings within previous reports showing that patients were primarily in their 50–60 s (Wakai et al., 1981; Fraioli et al., 1990; Onesti et al., 1990; McFadzean et al., 1991; Jugenburg et al., 1995; Biousse et al., 2001; Dubuisson et al., 2007; Möller-Goede et al., 2011). The male/female ratio was more than 3:1 showing a similar gender preponderance with previously reported studies (Wakai et al., 1981; McFadzean et al., 1991; Bills et al., 1993; Chacko et al., 2002; Sibal et al., 2004; Dubuisson et al., 2007; Zhang et al., 2009; Möller-Goede et al., 2011; Sarwar et al., 2013; Bujawansa et al., 2014; Vargas et al., 2014).

The patients in our study presented with classic symptoms, including headache, visual disturbance, and nausea and vomiting, without any life-threatening situations. Similar to previous studies, patients with ischemic PA are more likely to experience a long course of progression and relatively mild symptoms than classic pituitary apoplexy (Semple et al., 2008; Wang et al., 2019). More than 50% (25/45) of the patients in our study experienced subacute or chronic progression of symptoms from the initial onset, including headache, visual disturbance, and limited ocular movement, which partly explains why 33 patients received delayed surgical intervention. Moreover, asymptomatic pituitary apoplexy was reported to be common in the other subtypes of pituitary apoplexy (Briet et al., 2015). Only one patient in our study presented with asymptomatic pituitary apoplexy, which proposes the hypothesis that ischemic PA may lead to a large tumor infarct area, thus significantly increasing intrasellar pressure.

Until now, the etiology of pituitary apoplexy is still not well understood. According to previous reports, the precipitating factors of pituitary apoplexy include pituitary irradiation, intracranial pressure change, head trauma, hormone therapy, pregnancy, diabetes mellitus, diabetic ketoacidosis, cerebral angiography, anticoagulant drugs, pituitary function stimulation test, blood dialysis, and surgery (Briet et al., 2015). In addition, 31.43% (11/46) of patients in this study had at least one of the predisposing factors, including diabetes mellitus, hypertension, and anticoagulant therapy. These precipitating factors were similar to classic pituitary apoplexy. Moreover, 23.91% (11/46) of patients had focal ischemia or infarcted lesions on MRI. The manifestations of cerebrovascular fragility suggest that vascularization properties may play an important role in ischemic PA. Generally, due to the limited reported case of ischemic PA, it is challenging to determine the specific predisposing factors of ischemic PA.

The pathophysiology of ischemic PA remains unclear, but unique vascularization properties may contribute to its etiology. Based on the previous research, the possible mechanisms are as follows: (1) The rapid growth of tumors exceeds the angiogenesis supply (Oldfield and Merrill, 2015); (2) pituitary tumors have less vascular supply (Jugenburg et al., 1995; Turner, 2001); (3) tumor compression leads to increased intrasellar pressure (Rovit and Fein, 1972); (4) the decrease of systemic blood pressure leads to the decrease of blood supply (Biousse et al., 2001; Elsässer Imboden et al., 2005; Möller-Goede et al., 2011); (5) the fragility of blood vessels and vascular embolisms (Biousse et al., 2001). Of note, these factors may act together instead of singularly. As mentioned previously, patients in this cohort have a high rate of ischemia or infarcted lesions on MRI, which convinced us that the fragility of the blood vessels may be the primary cause of ischemic infarction. Coagulative necrosis of PA was generally considered the result of ischemic infarction (Xiao et al., 2015; Wang et al., 2019). However, Chacko et al. (2002) considered coagulative necrosis to be a late pathological manifestation of hemorrhagic infarction after an extended time interval between the acute onset and surgery (>8 weeks), which is obviously inconsistent with the findings of our study and other research (Nishioka et al., 2005; Kim et al., 2008; Xiao et al., 2015; Ogawa et al., 2016; Wang et al., 2019).

Imaging manifestations correspond to the underlying pathophysiology of ischemic PA. MRI is an effective imaging method for the diagnosis of pituitary apoplexy. The diffusion-weighted imaging (DWI) sequence of MRI can detect the infarcted area in a short time after tumor infarction (Rogg et al., 2002). Unfortunately, DWI is not routinely used in our patients. The MRI appearance of hemorrhagic PA was different at each stage (Dubuisson et al., 2007; Vaphiades, 2017; Waqar et al., 2017; Goyal et al., 2018). At the acute phase (7 days), the tumor showed iso- to hypointensity on T1WI and hypointensity on T2WI. During the subacute phase (7–14 days), hyperintensity on T1WI and T2WI can be observed. During the chronic phase (> 14 days), hypointensity on T1WI and T2WI can be observed. The MRI manifestations in our study are similar to previous research (Xiao et al., 2015; Ogawa et al., 2016; Wang et al., 2019). The tumor presented with iso- or hyperintensity on T1W and T2WI without enhancement after gadolinium injection regardless of what phase they were in. Rim enhancement of the tumor on MRI after gadolinium administration is a unique appearance for ischemic PA and was seen in 87.5% (35/40) of the patients in our study. It has been reported that rim enhancement, the outermost portion of the infarcted pituitary adenoma, was found to be the presence of granulation tissue and lymphocytosis based upon histological examination (Kleinschmidt-DeMasters and Lillehei, 1998). To our knowledge, rim enhancement, known as the pituitary ring sign, can also be seen in several pituitary diseases, such as craniopharyngioma, lymphocytic hypophysitis, and pituitary abscesses (Rogg et al., 2002; Arita et al., 2006; Zhu et al., 2019). Considering the presentation and imaging characteristic of pituitary apoplexy, the diagnosis of pituitary adenoma can be accurately made in sellar region diseases. Similar to typical pituitary apoplexy, thickening of the sphenoid sinus mucosa can also be seen in the majority of patients in this study and is likely attributed to increased pressure in the venous drainage system within the sinus area; thus, it is an indirect result of the increased intrasellar pressure (Liu and Couldwell, 2006; Briet et al., 2015; Vaphiades, 2017). Generally, MRI manifestations of ischemic PA have several unique characteristics corresponding to the histopathological findings, which are conducive to an accurate diagnosis before surgery (Semple et al., 2006, 2008; Wang et al., 2019).

Imaging features and intraoperative findings were the primary manifestations of the underlying pathophysiology of ischemic PA. During surgery, a yellow cottage cheese–like tissue was often observed (52.17%, 24/46). Similar to previous studies, the tumor in this study presented as yellow-white or yellow-grayish, with soft or uneven texture and poor-medium blood supply (Ogawa et al., 2016; Wang et al., 2019). Histological examination revealed massive coagulative necrosis with no intact adenoma cells. Ghost cells, with only ghost outlines and an acellular structure, were considered a unique manifestation of the pathological diagnosis of coagulative necrosis (Semple et al., 2008; Wang et al., 2019). It has been reported that the cottage cheese–like tissue, which may appear as iso- to hyperintensity on T1WI and non-enhancement on MRI after gadolinium injection, is in accordance with massive coagulative necrosis that has no intact adenoma cells under the light microscope (Wang et al., 2019).

Despite the advances in neurosurgical techniques and neurointensive care, there is still a lack of agreement regarding the best management of pituitary apoplexy. Supportive treatment and hormone replacement are essential for preoperative care and endocrine dysfunction. In this study, patients with hyponatremia, low serum cortisol, and secondary hypothyroidism received supportive therapy.

Four patients presented with headache and transient double vision; all patients received medical therapy and watchful observation. Surgical intervention was finally performed due to visual disturbance onset. Some studies have revealed an increasing role for conservative therapy in select cases, which often did not experience visual disturbance (Maccagnan et al., 1995; Ayuk et al., 2004; Gruber et al., 2006; Bujawansa et al., 2014; Singh et al., 2015). Five large retrospective studies compared the outcomes of conservative therapy and surgical treatment of patients with PA, and found that conservative treatment was able to achieve acceptable outcomes compared with surgical treatment (Ayuk et al., 2004; Sibal et al., 2004; Gruber et al., 2006; Leyer et al., 2011; Bujawansa et al., 2014). Although selection bias cannot be ignored that patients who received conservative treatment may have less severe visual dysfunction, therefore, pituitary apoplexy is increasingly considered to be a uniform diagnosis. For mild symptoms and subacute onset, conservative therapy should be more considered.

The surgical practice reflected the high rate of visual disturbance in our study. Moreover, 93.48% (43/46) of patients presented with visual loss and/or ocular palsy, which prompted the patients to receive surgical treatment. Four patients had almost no light perception. Neuro-ophthalmic outcomes were satisfactory that all patients obtained partial or complete remission, which was consistent with previous reports (Semple et al., 2006, 2008; Wang et al., 2019). However, there was no significant difference in ophthalmological outcomes between the early and the delayed surgery groups (Table 2). Furthermore, except for one patient who had slowly progressing visual loss and received early surgery due to sudden onset of headache, the rest of the patients obtained a partial visual recovery in the delayed surgery group. In addition, although Table 2 showed that there were no significant differences in preoperative baseline characteristics between the early and the delayed surgery groups, the incidence of visual loss was higher in the early surgery group. There is a possibility that patients in the early surgery group may have more severe ophthalmological dysfunction and therefore achieves a more favorable prognosis similar to that within the delayed surgery group. Moreover, there is a lack of a good evaluation system for preoperative ophthalmological dysfunction. Using Pituitary Apoplexy Score system (Rajasekaran et al., 2011), mild bilateral visual acuity impairment would obtain a higher score than the patients with unilateral blindness. The patients with unilateral blindness were more severe and more likely to receive early surgical intervention. Several studies reported that early surgical intervention did not show any statistically significant differences in the visual outcomes compared with the delayed surgery group (Bujawansa et al., 2014; Giritharan et al., 2016; Rutkowski et al., 2018). However, the selection bias in these studies cannot be ignored. Patients with severe situations would be more likely to undergo early surgical intervention. In contrast, several studies revealed that early surgical intervention could significantly improve visual outcomes (Bills et al., 1993; Randeva et al., 1999; Woo et al., 2010; Seuk et al., 2011). Generally, surgical intervention can achieve marked decompression and obtain satisfactory visual outcomes. Although the effect of surgical timing is still in the debate, early surgery should be advocated and seems to have a better visual outcome in this study.

Acute endocrine dysfunctions are prevalent in PA at the onset. It is reported that up to 80% of patients developed partial or panhypopituitarism (Rajasekaran et al., 2011). Our study reported a higher incidence of partial pituitary dysfunction, up to 91.30% (42/46). Similar to classic pituitary apoplexy, pituitary dysfunction showed no significant improvement regardless of the timing of surgical intervention (Table 2; Sibal et al., 2004; Gruber et al., 2006; Bujawansa et al., 2014; Singh et al., 2015; Giritharan et al., 2016; Rutkowski et al., 2018). Sixty-five percent of patients still received hormone replacement therapy at the last follow-up. Arafah et al. (1990) reported that the cases with pituitary dysfunction of classic PA recovered after surgical treatment. However, this study only included eight patients, a small sample size that does not provide strong recommendations. Surgery rarely leads to new pituitary dysfunction for patients without preoperative pituitary dysfunction. In general, endocrine dysfunction has shown no improvement following surgical intervention, and most patients still need long-term hormone replacement therapy postoperatively.

Patients in this study all obtained satisfactory curative effects after TSS without any complications. The total tumor resection rate was significantly higher in the early surgery group than in the delayed surgery group (*p* = 0.009, continuity correction) (Table 2). This result may be attributed to the high rate of invasiveness among the patients who underwent early surgery (*p* = 0.049, Pearson χ^2^) (Table 2). As mentioned previously, a tumor with more invasiveness leads to an increase of intrasellar pressure; thus, it is prone to experiencing pituitary apoplexy (Rovit and Fein, 1972). Therefore, periodic follow-up is necessary for the management of the disease. In summary, conservative therapy is suitable for selective patients; however, watchful observation is still necessary. Even during the long course of symptom onset, surgical intervention can achieve satisfactory outcomes regardless of surgical timing. The main strengths of our study are the large sample size of patients with ischemic PA, detailed clinical data, prognostic information, and prolonged follow-up. However, this study has limitations like all retrospective studies including selection bias and missing data. Two patients were lost during the follow-up. MRI examination was not implanted for four patients and partial endocrine information was missed in several patients. Besides, some important clinical factors are not available due to the limit of retrospective nature, like Ki-67. Furthermore, quite a few patients did not receive timely surgical decompression. Possible reasons are as follows: (1) Patients with chronic symptom onset are more likely to receive delayed surgery due to relatively mild symptoms and long onset duration; (2) many patients are transferred from other tertiary hospitals, which is a time-consuming process. A larger prospective multicenter controlled study and experimental investigation should be conducted to elucidate the natural history and pathogenesis of ischemic PA as well as formulate guidelines for the management of ischemic PA.

## Conclusion

Patients with ischemic PA can be accurately diagnosed by typical imaging characteristics preoperatively. The timing of surgical intervention does not significantly affect the resolution of neurological and endocrinological dysfunctions. Preoperative endocrine dysfunctions are common and usually appear to be poor after surgical intervention.

## Data Availability Statement

The raw data supporting the conclusions of this article will be made available by the authors, without undue reservation.

## Ethics Statement

The studies involving human participants were reviewed and approved by the Institutional Review Board of Beijing Tiantan Hospital. The patients/participants provided their written informed consent to participate in this study. Written informed consent was obtained from the individual(s) for the publication of any potentially identifiable images or data included in this article.

## Author Contributions

QZ and LW: study concept and design. QZ, YcL, ZF, and YkL: data acquisition and analysis. QZ and CZ: formal analysis and investigation. QZ: writing—original draft preparation. YcL, ZF, and YW: writing—review and editing. HZ, TL, JY, YZ, and YW: resources. LW: supervision and funding acquisition. All authors contributed to the article and approved the submitted version.

## Conflict of Interest

The authors declare that the research was conducted in the absence of any commercial or financial relationships that could be construed as a potential conflict of interest.

## Publisher’s Note

All claims expressed in this article are solely those of the authors and do not necessarily represent those of their affiliated organizations, or those of the publisher, the editors and the reviewers. Any product that may be evaluated in this article, or claim that may be made by its manufacturer, is not guaranteed or endorsed by the publisher.

## Abbreviations

PA: pituitary apoplexy
TSS: transsphenoidal surgery
TSH: thyroid-stimulating hormone
GH: growth hormone
IGF: insulin-like growth factor
LH: luteinizing hormone
FSH: follicle-stimulating hormone
DI: diabetes insipidus
ACTH: adrenocorticotropic hormone
FPA: functioning pituitary adenoma
NFPA: non-functioning pituitary adenoma
DWI: diffusion-weighted imaging.
